# Childhood B-acute lymphoblastic leukemia: a genetic update

**DOI:** 10.1186/2162-3619-3-16

**Published:** 2014-06-13

**Authors:** Jennifer S Woo, Michael O Alberti, Carlos A Tirado

**Affiliations:** 1 Department of Pathology and Laboratory Medicine, David Geffen School of Medicine, University of California, Los Angeles, 1010 Veteran Ave, 2nd Floor, room 2212 F, Los Angeles, CA 90024, USA

**Keywords:** Pediatric B-ALL, B-precursor, B-cell, Genetics, Cytogenetics

## Abstract

In the pediatric population, B-acute lymphoblastic leukemia (B-ALL) is the most prevalent childhood hematological malignancy, as well as the leading cause of childhood cancer-related mortality. Advances in cytogenetics utilizing array-based technologies and next-generation sequencing (NGS) techniques have revealed exciting insights into the genetic basis of this disease, with the hopes of developing individualized treatment plans for affected children. In this comprehensive review, we discuss our current understanding of childhood (pediatric) B-ALL and highlight the most recent genetic advances and their therapeutic implications.

## Introduction

ALL is a malignant clonal proliferation of lymphoid progenitor cells, most commonly of the B-cell lineage (B-ALL). In the pediatric population, ALL accounts for 81% of childhood leukemias; leukemia overall accounts for one third of cancers diagnosed in children between ages 0–14 years [1]. In the United States, the majority of ALL cases occur in ages 1–4, with an incidence rate in this group of 8 per 100,000, and preponderance for males over females [1]. The long-term rates of event-free survival (EFS) for childhood B-ALL approach 90%, although infants are associated with poorer prognosis and lower EFS rates [2-4].

This review discusses our current understanding of childhood B-ALL and highlights recent genetic advances and their therapeutic implications. Genetic classification of B-ALL is paramount for risk stratification and in treatment evaluation, especially within the context of clinical trial enrollment. At the forefront of pediatric oncologic research is the Children’s Oncology Group (COG) in North America, as well as the International Berlin-Frankfurt-Münster (BFM) Study Group in Europe, whose work has played a significant role in disease-specific research and therapeutic developments. Treatment protocols, including clinical trials, are the mainstay treatment for children with hematological and solid tumor malignancies. Cytogenetics advances of B-ALL have led to the discovery of numerous additional genetic changes, including mutations involving key cellular pathways in lymphoid development, tumor suppression, and cell cycle regulation. Uncovering the prognostic significance of these genetic aberrations is fundamental for risk stratification and ultimately individualized treatment.

### Clinical presentation

ALL is a disease of the bone marrow. Clinical suspicion for ALL arises with signs and symptoms reflective of bone marrow failure (pancytopenia) and/or extramedullary disease. The most common symptoms include fatigue, pallor, bone pain, arthritis, limping, easy bruising/bleeding, and/or petechiae. Physical examination may show lymphadenopathy and hepatosplenomegaly [5]. Extramedullary involvement may be seen in the central nervous system (CNS) with signs of increased intracranial pressure including headache [6]. In boys, testicular involvement may present as a testicular mass. Adverse prognostic factors include high leukocyte count on presentation, age <1 year or >10 years, and adverse cytogenetics [7,8].

### Histology

**Figure 1:**
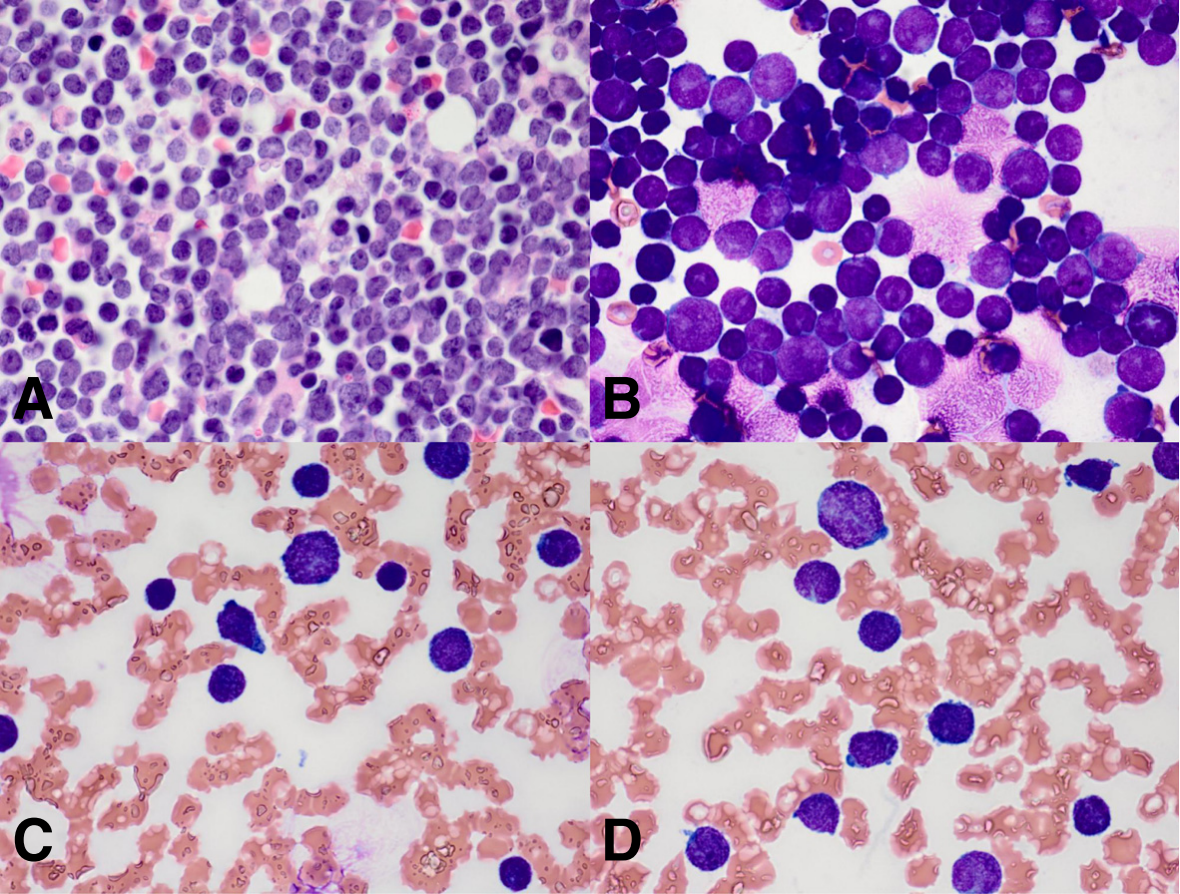
**Evaluation of a boy with abdominal pain, night sweats, increased fatigue, petechiae, and a white blood cell count of 113 × 10**^**3**^**/μL. A**. Bone marrow core biopsy (100×) showed diffuse replacement of normal marrow elements by uniform sheets of round to oval lymphoblasts with indented to convoluted nuclei. **B**. Touch preparation of core biopsy material showed lymphoblasts with high nuclear to cytoplasmic (N:C) ratio, finely dispersed nuclear chromatin, and prominent nucleoli. **C-D**. Peripheral blood smear (100×) showed lymphoblasts with high N:C ratio and cytoplasmic pseudopods.

Bone marrow aspirate studies show a preponderance of lymphoblasts with high nuclear to cytoplasmic (N:C) ratio, finely dispersed nuclear chromatin, and prominent nucleoli. On occasion, vacuolated cytoplasm and cytoplasmic pseudopods can be seen. Bone marrow core biopsies demonstrate diffuse replacement of normal marrow elements by uniform sheets of round to oval lymphoblasts with indented to convoluted nuclei [9] (Figure 1). No lower limit of blast percentage is required to establish the diagnosis, but most treatment protocols define ALL by the presence of greater than or equal to 25% blasts. By immunophenotype, B-lymphoblasts demonstrate universal positivity for B-cell markers including CD19, cytoplasmic CD79a, and cytoplasmic CD22; as well as positivity for surface CD22, CD24, PAX5 and TdT; with variable expression of CD10, CD20 and CD34 [9]. CD10 is often negative in infant ALL (age <1 year) with *MLL* (11q23) gene rearrangements [10].

### Recurrent genetic abnormalities

Approximately 75% of childhood ALL cases harbor recurrent genetic abnormalities, including aneuploidy or structural chromosomal arrangements, detected by conventional karyotyping and fluorescence *in situ* hybridization (FISH) [11]. Translocations t(9;22)(q34;q11) [*BCR*-*ABL1*], t(12;21)(p13;q22) [*ETV6*-*RUNX1* (*TEL*-*AML1*)], hyperdiploidy, and translocation t(4;11)(q21;q23) [*MLL*-*AFF1*(*AF4*)] in infants, are found at the highest frequency in childhood B-ALL [11]. Other recurrent cytogenetic abnormalities include hypodiploidy and translocation t(1;19)(q23;p13) [*TCF3*-*PBX1* (*E2A*-*PBX1*)] [11]. Advances in cytogenetics utilizing array-based technologies and NGS have uncovered additional submicroscopic DNA alterations affecting genes involved in normal hematopoiesis, tumor suppression, apoptosis, and cell cycle regulation, including *IKZF1*, *CRLF2*, *PAX5*, and *FLT3* (Table 1). Advanced techniques have revealed new insights into well-known recurrent abnormalities, and have more importantly elucidated new gene targets involved in aberrant hematopoiesis and relapse. Overall, the utilization of these newly identified genetic alterations has clinical utility for diagnosis, risk stratification, and targeted therapy.

**Table 1:** Recurrent genetic abnormalities in B-ALL, associated affected genes, and prognosis

**Recurrent genetic abnormality**	**Common genes implicated**	**Prognosis**	**Additional comment**	**References**
**Aneuploidy**				
High-hyperdiploidy		Good	*FLT3* mutations can be seen in hyperdiploid B-ALL. Almost 80% of cases display further genetic abnormalities of no definitive clinical significance.	[14,93]
Hypodiploidy		Poor		
Near-hypodiploidy			Concomitant alterations in RTK- and Ras-signaling (*NF1*), as well as *IKZF3* (*Aiolos*) may be seen.	[22]
Low-hypodiploidy			Concomitant alterations in *TP53*, *RB1*, *IKZF2* (*Helios*) may be seen.	[22]
**Recurrent translocations**				
t(12;21)(p13;q22)	*ETV6*-*RUNX1* (*TEL*-*AML1*)	Good		
t(1;19)(q23;p13)	*TCF3-PBX1* (*E2A*-*PBX1*)	Intermediate		
t(9;22)(q34;q11)	*BCR-ABL1* (Philadelphia chromosome; Ph+)	Intermediate	Associated with older age, higher leukocyte count, and more frequent CNS leukemia at time of diagnosis.	[34]
*MLL* (11q23) rearrangements		Poor	Almost exclusively seen in infant B-ALL. *FLT3* mutations are often seen with *MLL* rearrangements. Epigenetic aberrancies, through microRNAs, are implicated in the pathogenesis of *MLL*-rearranged B-ALL.	[93,98]
t(4;11)(q21;23)	*MLL-AFF1*(*AF4*)			
t(9;11)(p22q23)	*MLL-MLLT3*(*AF9*)			
t(11;19)(q23;p13.3)	*MLL-ENL*			
t(10;11)(p13-14;q14-21)	*MLL-MLLT10*(*AF10*)			
**Additional genetic alterations**				
*BCR-ABL1*-like ALL	*IKZF1*, *CRLF2*, *JAK* mutations	Poor	Defined by a similar GEP to Ph + B-ALL, but in the absence of the *BCR-ABL1* rearrangement [t(9;22)]. Rearrangements in *CRLF2* or *EBF1*-*PDGFRB*, as well as concurrent *JAK* mutations, and/or *IKZF1* (*Ikaros*) deletions/mutations may be seen.	[60,61,89]
*JAK* mutations, including *JAK2* (9p24)	*IKZF1*, *CRLF2*, *CDKN2A/B* (p16)	Poor	In the setting of *BCR-ABL1*-like B-ALL, *JAK* mutations are associated with concomitant *IKZF1* (*Ikaros*) and *CDKN2A/B* (p16) alterations. *JAK2* mutations are also associated with *CRLF2* rearrangements, and have been described in 60% of Down syndrome-associated ALL.	[72-78]
iAMP21	*RUNX1*, *P2RY8-CRLF2*	Poor	Occurs in older children with B-ALL. Associated with *P2RY8-CRLF2*, resulting in the overexpression of *CRLF2*.	[56,57]
*IgH@* (14q32) rearrangements	*IgH@* with multiple fusion partners	Poor	Occurs in older children, adolescents, and young adults. Recurrent fusion partners include *CRLF2*, *ID4*, *CEBP*, and *EPOR.*	[46,51]
*FLT3* (13q12) mutations	*FLT3*	Poor	Seen in MLL-rearranged and hyperdiploid B-ALL.	[93,94]
*PAX5* (9p13) rearrangements, deletions	*PAX5* with multiple fusion partners	Unknown	Reported rearrangements with multiple genes, including *ETV6* and *JAK2.*	[75,91,92]
**Relapsed all**	*CDKN2A/B*, *ETV6*, *IKZF1*, *CREBBP*, *NT5C2*	Poor	20% of total pediatric ALL relapse cases and 60% of high-hyperdiploid relapse cases harbor mutations in *CREBBP.*	[103,104,106-109,111]

### Aneuploidy

#### ***High hyperdiploidy***

**Figure 2:**
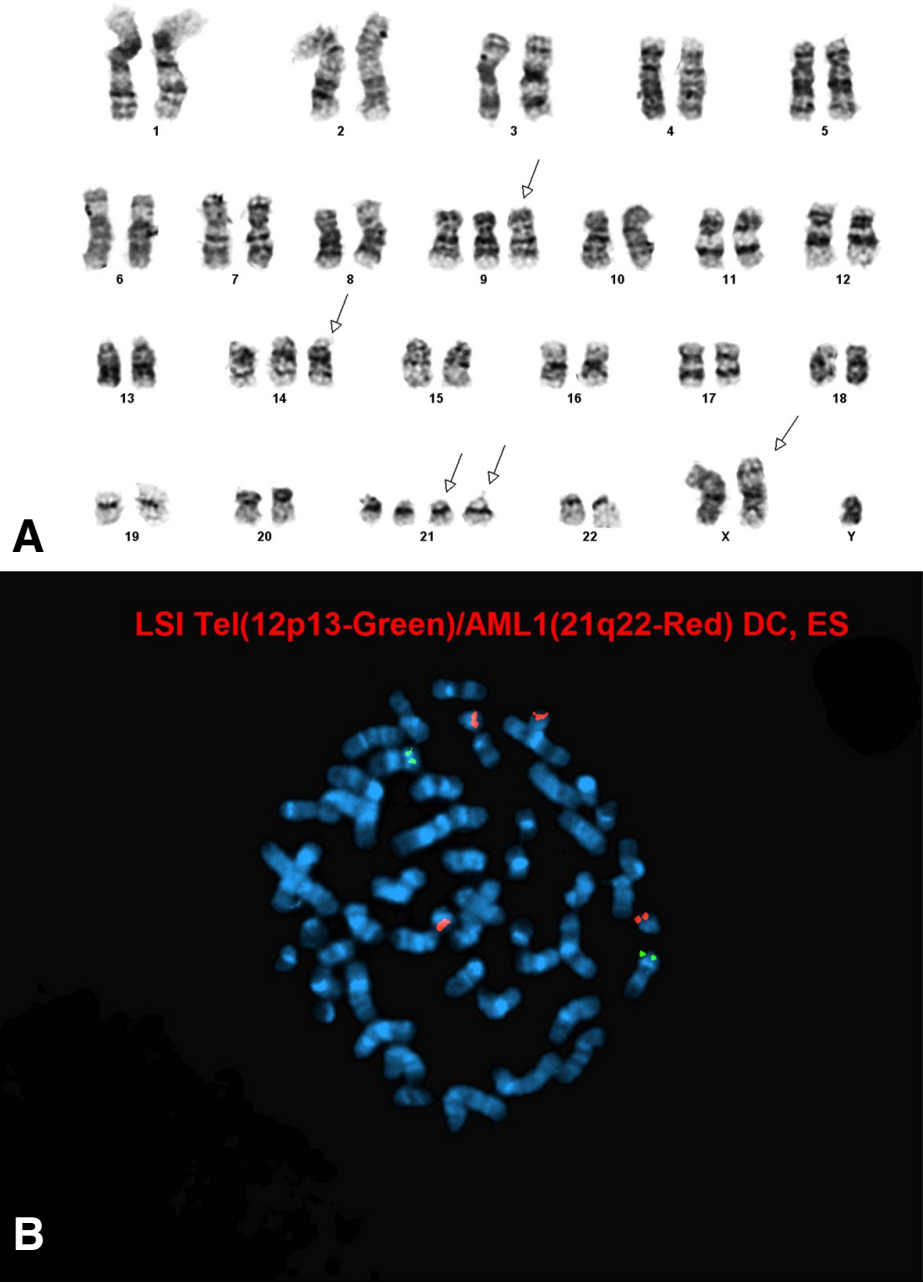
**Evaluation of a 3 year-old boy with hyperdiploid B-ALL. A**. Abnormal male hyperdiploid karyotype with extra copies of chromosomes X, 2, 9, 14. **B**. FISH analysis detected +9q, +14q and +21q in 96% , 93.7% and 96% of the nuclei examined, respectively. In addition, 12p deletion was observed in 89% of the nuclei examined, suggestive of an underlying complex aneuploid (most likely hyperdiploid) karyotype.

High hyperdiploidy (51–65 chromosomes) is one of the most common cytogenetic abnormalities observed in childhood B-ALL (Figure 2). It is seen in 25-30% of total childhood B-ALL cases, with the highest frequency in the 1 to 4 year age range [12,13]. High hyperdiploidy is characterized by a nonrandom gain of chromosomes, including + X, +4, +6, +10, +14, +17, +18, and +21 [13,14]. The diagnosis confers a good prognosis in childhood B-ALL, with EFS rates of approximately 80% and overall survival (OS) rates of 90% (reviewed in [13]). Despite favorable outcomes, 20% of children relapse and 10% eventually succumb to the disease [15]. SNP array analysis of high hyperdiploid B-ALL has shown that almost 80% of cases display further genetic abnormalities in addition to characteristic chromosomal gains; although these additional aberrations have no definitive clinical ramification [14].

Recently, rare alleles of *PRDM9* (which encodes a meiosis-specific histone H3 methyltransferase that controls activation of recombination hotspots) have been reported to be associated with the development of high hyperdiploid and infant B-ALL [16,17]. Furthermore, it was even postulated that PRDM9 activity during the early stages of meiosis in the parental germline could lead to genomic instability and development of childhood B-ALL [16].

#### ***Hypodiploidy***

Hypodiploidy is characterized by fewer than 46 chromosomes and is seen in 5-8% of total B-ALL cases [18,19]. The current high risk COG protocol AALL1131 denotes hypodiploidy as less than 44 chromosomes. The majority of hypodiploid B-ALL contain 45 chromosomes. The remainder of hypodiploidly cases are much rarer and include high-hypodiploid (40–44 chromosomes), low-hypodiploid (33–39 chromosomes), and near-haploid (24–29 chromosomes) groups [19,20]. In general, hypodiploidy with less than 40 chromosomes confers a poor prognosis. The 3-year EFS for near-haploid and low-hypodiploid B-ALL is 30% [19,20]. Hypodiploid cases have also been shown to undergo reduplication, resulting in a hyperdiploid karyotype (so called “masked hypodiploid ALL”), which may preclude the correct genetic classification and therefore render an inappropriate treatment regimen [20,21].

A recent genomic profiling study of hypodiploid ALL cases identified multiple recurrent genetic alterations, distinguishing near haploid from low-hypodiploid ALL [22]. Near-haploid ALL cases showed alterations targeting genes in receptor tyrosine kinase (RTK) signaling and Ras signaling (*NF1*) pathways, as well as high frequency alterations in the IKAROS gene family, particularly *IKZF3* (*Aiolos*) which encodes for the zinc finger transcription factor AIOLOS [22]. Low-hypodiploid cases showed genetic alterations of *TP53*, *RB1*, and *IKZF2* (*Helios*) [22]. In the *TP53* mutated cases, non-tumor cells also harbored *TP53* mutations, suggesting an inherited basis of disease and a possible manifestation of Li-Fraumeni syndrome (LFS) [23]. Both low-hypodiploid and near-haploid ALL showed activation of Ras-signaling and PI3K (phosphoinositide 3-kinase)-signaling pathways that were sensitive to PI3K inhibitors such as rapamycin in vitro, suggesting that PI3K inhibitors could be explored as a therapeutic treatment option [22].

### Recurrent translocations

#### ***ETV6-RUNX1 (TEL-AML1)***

**Figure 3:**
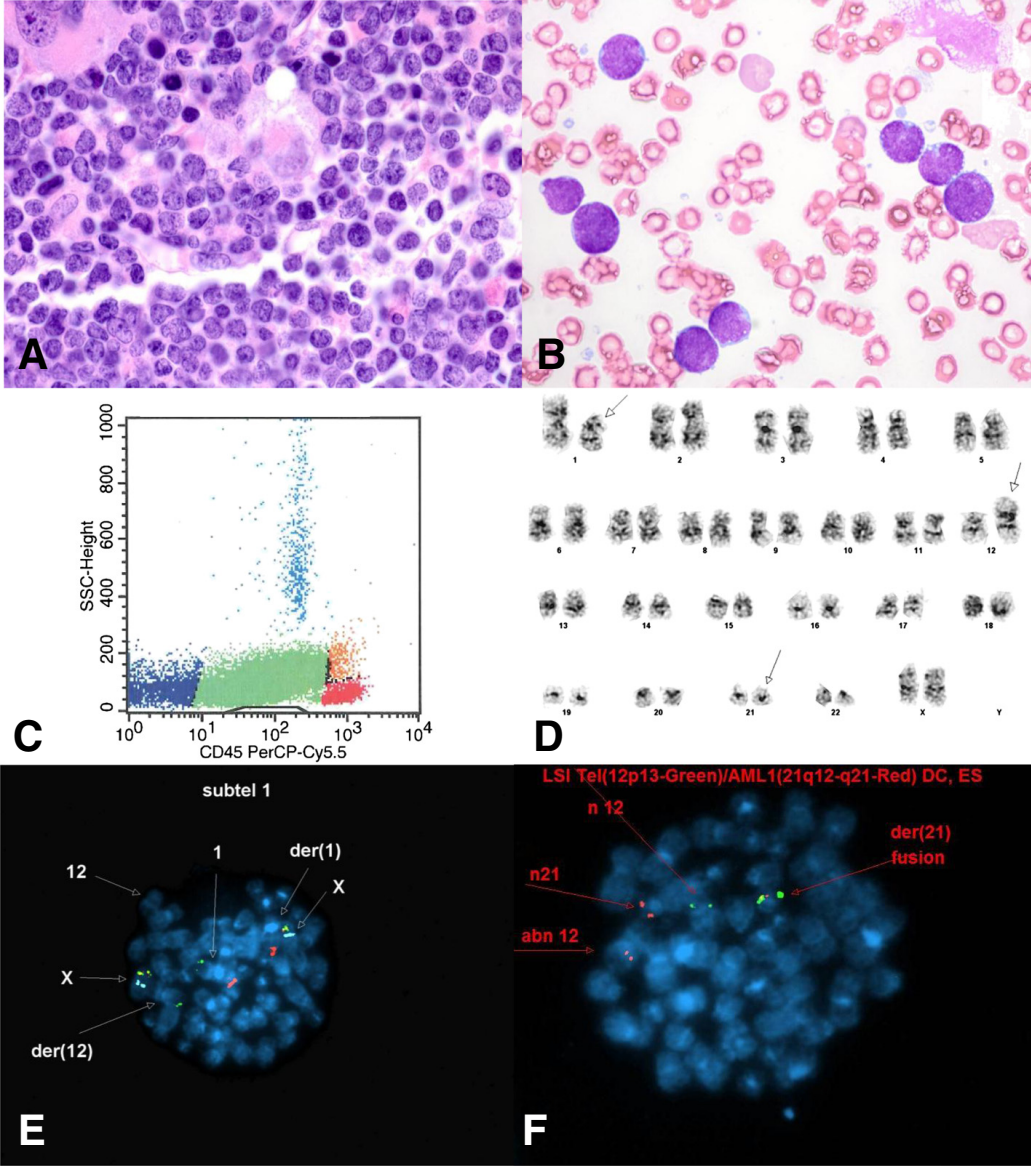
**Evaluation of a 2 year-old girl presenting with fevers. A**. Bone marrow core biopsy (100×) showing sheets of round to oval lymphoblasts. **B**. Bone marrow aspirate (100×) showing lymphoblasts with cytoplasmic vacuoles. **C**. Representative flow cytometry histogram. The CD45(dim) gated population contained excess B-lymphoblasts (81% of total), positive for CD10, CD19, CD34, CD38, CD79a, HLA-DR, and TdT. **D**. Abnormal female karyotype with unbalanced rearrangements of 1p, a derivative chromosome 12 (due to an unbalanced translocation between chromosomes 1p and 12p), and a derivative chromosome 21 (due to an unbalanced translocation between chromosomes 12 and 21), resulting in *ETV6-RUNX1* fusion. **E-F**. Abnormal FISH signal pattern consistent with *ETV6-RUNX1* (*TEL-AML1*) fusion, indicative of t(12;21) translocation.

**Figure 4:**
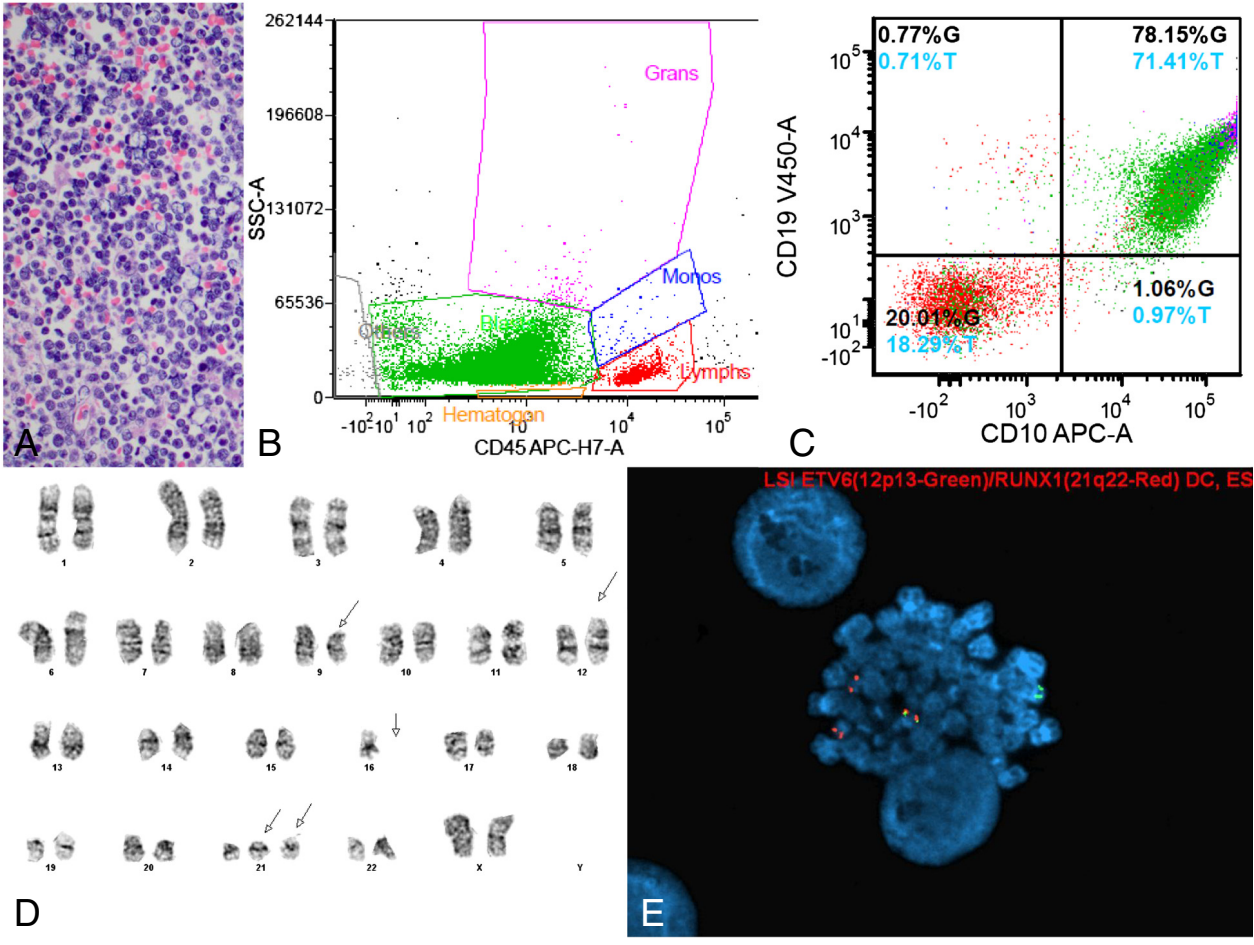
**Evaluation of a 3 year-old girl with pancytopenia. A**. Bone marrow core biopsy (40×) showing sheets of lymphoblasts. **B-C**. Representative flow cytometry histograms. The CD45(dim) gated population contained excess and abnormal B-lymphoblasts (85% of the total), positive for CD10, CD13, CD19, CD22, CD34, CD38, HLA-DR, plus intracellular CD79a, intracellular CD22, and TdT. **D**. Abnormal composite female karyotype with monosomy 16, trisomy 21, and deletions of 6q and 9q. **E**. FISH analysis detected the *ETV6*-*RUNX1* (*TEL*-*AML1*) fusion, indicative of t(12;21) translocation. In addition, 4.4% of these abnormal cells showed an extra copy of the *RUNX1* locus, suggestive of an underlying +21q.

**Figure 5:**
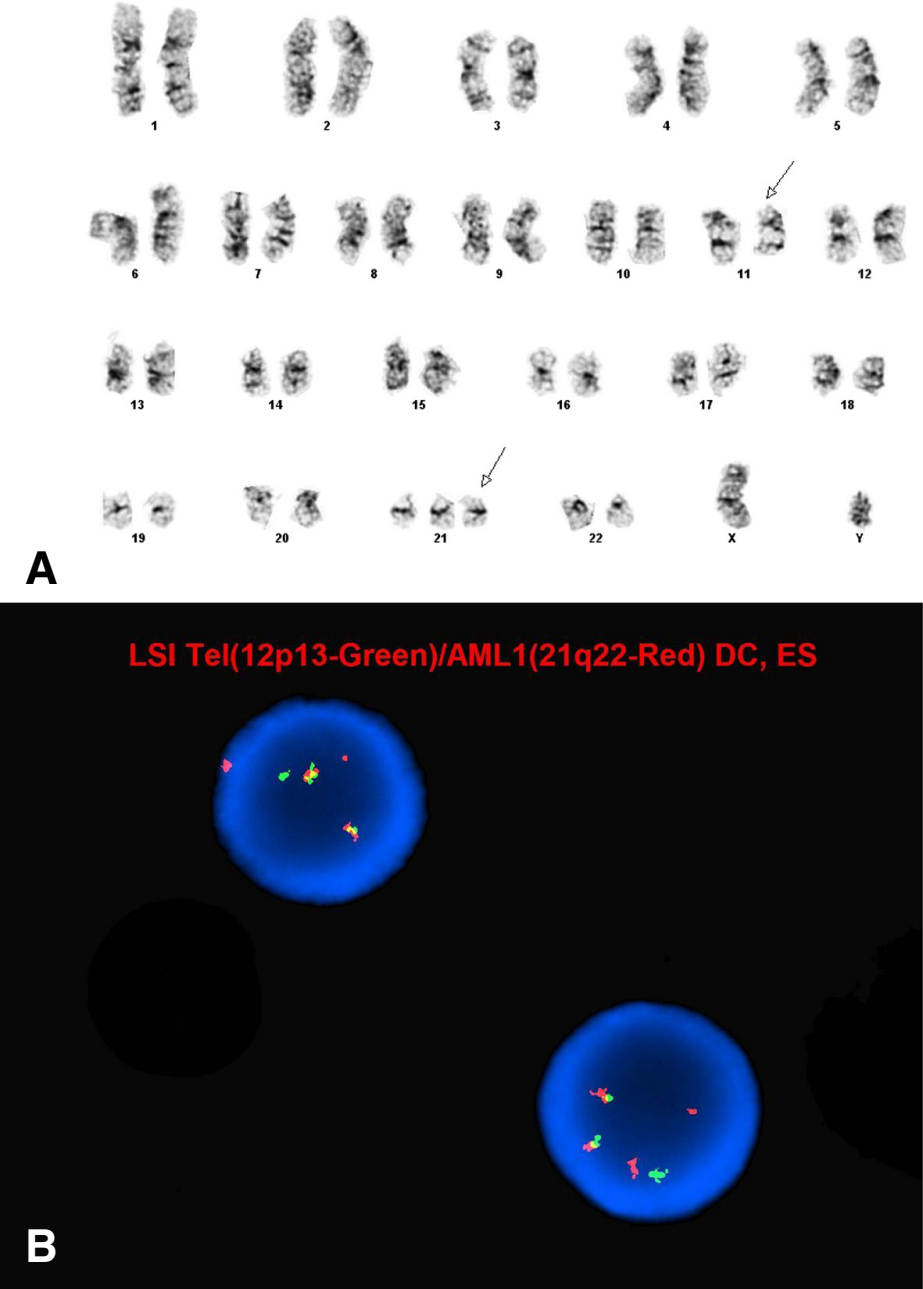
**Evaluation of a 7 year-old boy with B-ALL. A**. Abnormal male karyotype with a deletion of 11q and trisomy 21. **B**. FISH analysis demonstrated an abnormal signal pattern consistent with *ETV6*-*RUNX1* (*TEL*-*AML1*) fusion, indicative of t(12;21) translocation, as well as +21q and 11q- (*MLL* deletion).

The most common chromosomal rearrangement in B-ALL is t(12;21)(p13;q22), encoding for *ETV6*-*RUNX1* (*TEL*-*AML1*) [11] (Figures 3, 4 and 5). It occurs in 25% of children with B-ALL and confers an excellent prognosis [24,25]. Both ETV6 and RUNX1 transcription factors are required for normal hematopoiesis [26,27]. The ETV6-RUNX1 fusion protein is thought to disrupt the normal expression of RUNX1-regulated genes by converting RUNX1 to a transcriptional repressor [28].

#### ***TCF3-PBX1 (E2A-PBX1)***

**Figure 6:**
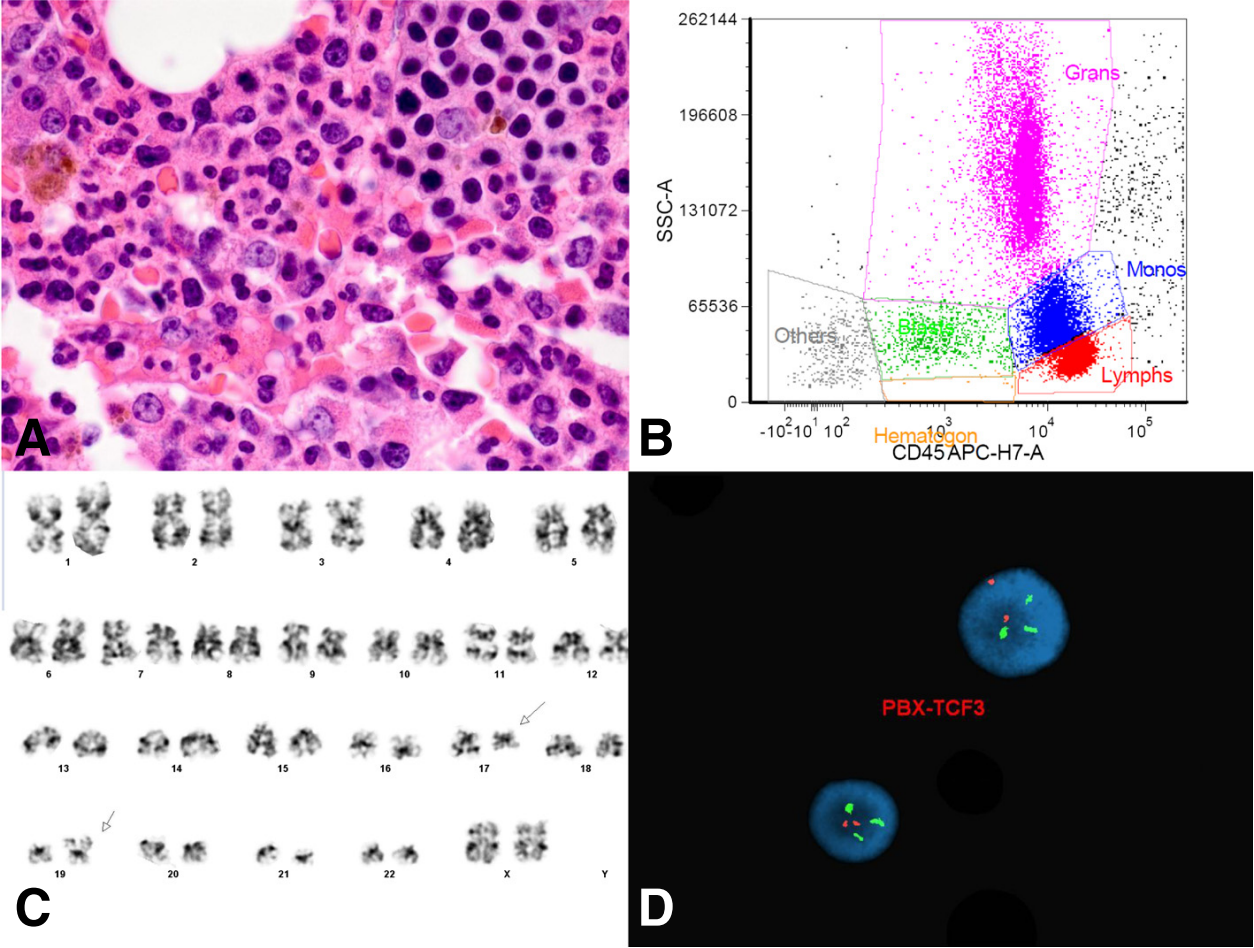
**Evaluation of an 18 year-old female with B-ALL. A**. Variably cellular marrow (100×) with clusters of B-lymphoblasts and reduced multilineage hematopoiesis. **B**. Representative flow cytometry histogram. The CD45(dim) gated population comprised approximately 4% of total cells and contained no excess blasts. **C**. Abnormal female karyotype demonstrating t(17;19) translocation. **D**. FISH analysis detected an abnormal signal pattern compatible with *TCF3* (19p13) rearrangement.

The t(1;19)(q23;p13) rearrangement and its unbalanced variant der(19)t(1;19)(q23;p13) are commonly seen in B-ALL [11] (Figure 6). The resultant TCF3-PBX1 (E2A-PBX1) fusion protein is comprised of the transactivation domains of TCF3 and a DNA binding domain of the homeobox protein PBX1, converting PBX1 into a transactivating factor and reducing expression of the *TCF3* encoded transcription factors E12 and E47, required for early lymphoid development [29,30]. The translocation occurs in 6% of childhood B-ALL and is historically associated with poorer outcomes [31]. However, advances in treatment have improved clinical outcomes of children with this abnormality and the translocation is now thought to confer an intermediate prognosis [32].

#### ***BCR-ABL1 (Philadelphia chromosome)***

B-ALL harboring the t(9;22)(q34;q11) translocation or ‘Philadelphia chromosome’ (Ph + B-ALL), encodes the fusion gene *BCR-ABL1.* This translocation is present in 3-5% of childhood B-ALL cases [33] and is associated with older age, higher leukocyte count, and more frequent CNS involvement at time of diagnosis [34]. The translocation fuses the 5’ sequence of the breakpoint cluster region (*BCR*) on chromosome 22 to the 3’ sequence of the *ABL1* gene on chromosome 9. The resultant oncoprotein is a constitutively active nonreceptor tyrosine kinase, responsible for leukemogenesis. The *BCR* region contains two breakpoint areas, including a major *BCR* (M-bcr) area commonly seen in chronic myelogenous leukemia (CML), and a minor *BCR* (m-bcr) area seen in pediatric B-ALL. In 90% of childhood B-ALL cases, fusion genes created by breaks in m-bcr encode for a 190 kDa fusion protein (p190) [35].

The use of ABL1 tyrosine kinase inhibitors (TKIs), such as imatinib, has been revolutionary in the treatment of Ph + B-ALL. Once associated with dismal outcomes, use of TKIs combined with intensive chemotherapy has improved 3-year EFS in children and adolescents with Ph + B-ALL with no appreciable increase in toxicity [36]. The COG AALL0622 clinical trial, substituting the second generation TKI, dasatinib, for imatinib, is under investigation [37].

#### ***MLL gene rearrangements***

*MLL* (mixed-lineage-leukemia) gene rearrangements at 11q23 are present in 80% of all infant B-ALL cases and 10% of all childhood B-ALL [38,39]. The *MLL* gene encodes for a protein with histone methyltransferase activity, which is essential for hematopoietic regulation of *HOXA* and *MEIS1* gene expression [40]. The most common gene rearrangements include t(4;11)(q21;q23) encoding *MLL-AFF1*(*AF4*), t(9;11)(p22;q23) encoding *MLL-MLLT3*(*AF9*), t(11;19)(q23;p13.3) encoding *MLL-ENL*, and t(10;11)(p13-14;q14-21) encoding *MLL-MLLT10*(*AF10*) [41,42]. About 50% of *MLL* rearrangements show the t(4;11)(q21;q23) translocation. In general, *MLL* rearrangements are associated with adverse outcomes, with an EFS of approximately 35% [43], largely due to cellular drug resistance [44,45].

#### ***IGH@ translocations***

Rearrangements of the immunoglobulin heavy chain locus (*IGH@*) on chromosome 14q32 are rare in B-ALL, occurring in <5% of cases [46]. *IGH@* rearrangements occur more frequently in adolescents and appear to have poor clinical outcomes. The most common *IGH@* partners include *CRLF2* (*cytokine receptor-like factor 2*) at the pseudoautosomal region 1 (PAR1) of Xp22.3/Yp11.3 (resulting in overexpression of *CRLF2*) [47], *ID4* (inhibitor of DNA binding 4) at 6p22 [48], and members of the *CEBP* (CCAAT/enhancer binding protein) family [49,50]. Translocations between *IGH@* and *EPOR* (erythropoietin receptor) at 19p13 have also been reported [51-53], with other remaining translocations appearing sporadic [46].

### Intrachromosomal amplification of chromosome 21 (iAMP21)

iAMP21 is defined as the presence of three or more copies of the *RUNX1* gene within a morphologically abnormal chromosome 21 [54,55]. Amplified regions on chromosome 21 are found within a 5.1 Mb region containing *RUNX1*, *miR-802*, and genes mapping to the Down syndrome critical region. iAMP21 occurs in approximately 2% of older children with B-ALL, and is associated with poorer outcomes when treated with standard therapy, and also increased risk for early and late relapse [56]. The five-year EFS is approximately 29%, with an OS of 71% [57]. Recent studies have shown that treatment of iAMP21 patients as high-risk provides a significant improvement in outcome [58]. One recurrent abnormality in iAMP21, seen in 35% of children with iAMP21, includes the *P2RY8-CRLF2* fusion (created by a focal deletion at the PAR1 region at Xp22.3/Yp11.3) which results in the overexpression of *CRLF2 *[47]. Gain of the X chromosome, and abnormalities affecting the genes *IKZF1* (*Ikaros*), *CDKN2A*, *PAX5*, *ETV6*, and *RB1* have also been associated with iAMP21 [56]. Lastly, individuals born with the constitutional Robertsonian translocation rob(15;21)(q10;q10)c have a 2700-fold increased risk of developing B-ALL with iAMP21 [59].

### Submicroscopic genetic alterations

#### ***The role of IKZF1 in BCR-ABL1 and BCR-ABL1-like ALL***

“*BCR-ABL1*-like” B-ALL, seen in 15% of childhood B-ALL, has recently been defined by gene expression profiling (GEP). *BCR-ABL1*-like B-ALL shows a similar GEP to Ph + B-ALL, but in the absence of the *BCR-ABL1* rearrangement [60,61]. These cases are associated with poor outcomes and increased relapse risk [62-64]. Deletions and sequence mutations in *IKZF1* (*Ikaros*) at 7p13, which encodes for the lymphoid transcription factor IKAROS, are associated with approximately 70% and 40% of *BCR*-*ABL*-positive and *BCR-ABL1*-like B-ALL, respectively [60,63]. Despite improvements in outcomes for children with Ph + B-ALL that has resulted from combination TKI and chemotherapy, recent studies have demonstrated that Ph + ALL harboring *IKZF1* deletions are associated with unfavorable outcomes; irrespective of imatinib treatment [65]. In addition to frequent *IKZF1* (*Ikaros*) abnormalities, up to 50% of *BCR-ABL1*-like ALL harbor rearrangements in *CRLF2*, with concurrent Janus kinase family (*JAK*) mutations [66,67]. *BCR-ABL1*-like ALL has also shown resistance to L-asparaginase, and to a lesser extent, daunorubicin; although intensified therapy could give more suitable treatment options [60].

Additional *BCR-ABL1*-like ALL studies have shed light on the heterogenous pathogenesis of ALL. In 2013, a genome-wide association study (GWAS) of *BCR-ABL1*-like ALL identified a susceptibility locus for *BCR-ABL1*-like ALL (*GATA3*, rs3824662) [68]. This locus was shown to be associated with *CRLF2* rearrangements, *JAK* mutations, and deletions in *IKZF1* (*Ikaros*) [68].

Transcriptome and whole-genome sequencing of *BCR-ABL1*–like ALL has also identified other genetic alterations involved in the activation of kinase signaling, including *EBF1-PDGFRB*, comprised of the transcription factor *EBF1* (early B-cell factor 1) and the receptor tyrosine kinase *PDGFRB* (platelet-derived growth factor receptor β), resulting from 5q33q33 microdeletion [53,69]. Several reports suggest that the use of TKIs to treat B-ALL harboring the *EBF1-PDGFRB* rearrangement may be of clinical benefit [70,71].

#### ***JAK mutations and CRLF2 rearrangements***

The role of cytokine receptors and *JAK* family members are playing increasingly larger roles in B-ALL studies. The *JAK* family encodes four nonreceptor tyrosine kinases (*JAK1*, *JAK2*, *JAK3*, *TYK2*) involved in cytokine-mediated signaling (JAK-STAT pathway) [72]. Mutations occur in about 10% of high-risk childhood B-ALL cases [73]. In the setting of *BCR-ABL1*-like B-ALL, *JAK* mutations are also associated with concomitant *IKZF1* (*Ikaros*) and *CDKN2A/B* alterations, and correlate with worse outcomes [74,75]. *JAK2* mutations are also associated with *CRLF2* rearrangements (as described above), and are described in 60% of Down syndrome (Trisomy 21)-associated ALL [76,77]. Approximately 40% of *CRLF2-*rearranged cases can harbor *JAK2* mutations [78].

Abnormalities involving *JAK2* (9p24) most often arise via point mutations involving the pseudokinase domain R683 [79,80]; however, rare cases of *JAK2* rearrangements have also been identified [79,81,82]. In *JAK2* rearrangements, dimerization or oligomerization of JAK2 is induced without ligand binding, resulting in constitutive activation of downstream pathways in leukemic cells. Alterations in *CRLF2*, (Xp22.3/Yp11.3), occur in up to 8% of unscreened childhood B-ALL cases and up to 15% of high-risk B-ALL patients [83-86]. *CRLF2* rearrangements result in constitutive activation of the STAT5 pathway, resulting in leukemogenesis. Additionally, abnormal PI3K/mTOR pathway signaling has also been implicated [87].

The JAK2 inhibitor, ruxolitinib, has been shown to reduce tumor burden in xenograft mouse models harboring *BCR*-*JAK2* [t(9;22)(p24;q11.2)] [88], and has demonstrated promising results in the treatment of *CRLF2*-rearranged*, JAK2*-mutated leukemic cells in vitro [87]. Additionally, the PI3K inhibitor, rapamycin, has been shown to control leukemic burden [88]. Clinical trial NCT01251965, utilizing ruxolitinib in refractory or relapsed ALL or AML (acute myelogenous leukemia), is currently ongoing.

More recently, another potential molecular target in *JAK2*-mutated B-ALL was revealed. In a mouse model, overexpression of mutant *JAK2* led to downstream upregulation of prosurvival *Bcl-2* gene family members, and combined use of the Bcl-2/Bcl-xL inhibitor ABT-737 with JAK2 inhibitors prolonged disease regression time [89].

#### ***PAX5 deletions and rearrangements***

*PAX5* (9p13), a member of the paired box gene family, is a transcription factor necessary for normal hematopoietic development [90]. In childhood B-ALL, mutations in *PAX 5* have been detected in 32% of cases by genome-wide analysis [75]. *PAX5* rearrangements occur with incidence of about 2.5%, with numerous reported rearrangements including *ETV6* (12p13) and *JAK2* (9p24) [91]. Recently, a heterozygous germline *PAX5* variant, c.547G > A, encoding p.Gly183Ser, was identified in two unrelated families with autosomal dominant B-ALL, suggesting that *PAX5* mutations may play a role in the inherited susceptibility of B-ALL [92].

#### ***FLT3 mutations***

*FLT3* (fms-tyrosine kinase 3) on chromosome 13q12 is frequently mutated in *MLL*-rearranged and high hyperdiploid B-ALL [93]. Infants with *MLL-*rearrangements have been shown to be sensitive to the FLT3 inhibitor, PKC412 (midostaurin), suggesting that multitarget kinase inhibition may present as novel therapeutic modalities [94]. COG study AALL0631, utilizing the FLT3 inhibitor lestaurtinib (CEP701) along with standard chemotherapy, is currently ongoing in infants with *MLL* rearrangements.

### The role of epigenetics

The role of epigenetic regulation in B-ALL has gained considerable attention. In 2013, the first integrated genome-wide analysis in childhood ALL, incorporating cytosine methylation profiling, DNA copy number alterations (CNA) and GEP, was reported [95]. Recurrent epigenetic alterations were identified across all B-ALL subtypes studied, suggesting that certain epigenetic events are required for leukemic transformation [95]. Moreover, genes frequently affected by structural abnormalities were shown to be targets for aberrant DNA methylation [95]. Additionally, global histone modification profiling revealed a distinct molecular chromatin signature in several ALL cell lines, subsequently noted to harbor abnormalities in *NSD2*, encoding for a methyltransferase [96]. Targeted investigation of patient samples revealed approximately 7.5% of childhood B-ALL harbored mutations in *WHSC1*/*NSD2* (particularly p.E1099K) but were enriched the B-ALL subtypes *ETV*-*RUNX1* (20%) and *TCF3*-*PBX1* (15%) [96]. More recently, NGS targeted exome profiling identified a number of epigenetic regulators, including *CREBBP* and *SETD2*, mutated in 25% of B-ALL samples at time of diagnosis [97]. Further analysis revealed that mutations in *SETD2* were enriched in *MLL*-rearranged (22%) and the *ETV*-*RUNX1* (13%) subtypes of B-ALL, and that over 50% of matched relapsed cases, regardless of subtype, demonstrated enrichment of mutations in epigenetic regulators (discussed more below) [97].

Epigenetic studies have also shown insights into our understanding of *MLL-*rearranged B-ALL. MicroRNAs (miRNAs), short noncoding RNAs involved in the regulation of signaling pathways of cell differentiation, proliferation, and apoptosis, have been shown to promote leukemogenesis through aberrant epigenetic activity [98]. The presence of these epigenetic aberrancies suggests that histone deacetylase (HDAC), DNA methyltransferase (DNMT), and/or histone methyltransferase (HMT) inhibitors may play a therapeutic role in *MLL*-rearranged B-ALL [99,100]. Notably, selective DOT1L HMT inhibitors, such as EPZ-004777, have been shown to selectively destroy *MLL*-rearranged cells in mouse models [101,102]. Clinical trial NCT01684150 is currently evaluating the use of the DOT1L HMT inhibitor, EPZ-5676, in adults with MLL-rearrangements.

### Relapsed ALL

Despite overall progress in treatment, relapsed B-ALL has a dismal prognosis with an overall survival of 30% [103]. Several genetic subtypes and aberrations are associated with high treatment failure risk, including *CDKN2A/B*, *ETV6*, and *IKZF1* (*Ikaros*) mutations. Relapse occurs across the spectrum of all B-ALL subtypes, with some cases demonstrating acquisition of additional chromosomal abnormalities over time. In 2008, genome-wide analysis of matched samples from diagnosis and relapse identified different patterns of genomic CNA for samples at diagnosis and relapse, with that acquired abnormalities preferentially affected cell cycle regulation and B-cell development genes [104]. They determined that only 8% of relapse clones carried identical CNA to the diagnostic clone, while almost 90% of relapse clones evolved to acquire additional or some but not all CNA from the diagnostic clone [104], highlighting that relapse in ALL is variable and complex, but often a descendent of an ancestral clone to the principal de novo leukemia clone (reviewed in [105]).

Gene sequencing studies in 2011 [106] and 2012 [107], demonstrated 20% of total pediatric ALL relapse cases [106] and 60% of high-hyperdiploid relapse cases [107] harbor mutations in *CREBBP,* a transcriptional co-activator and histone acetyltransferase (HAT). Mutations in *CREBBP* have been shown to affect the regulation of glucocorticoid responsive genes. Because glucocorticoids are the cornerstone of B-ALL therapy, *CREBBP* mutations are thought to influence response to treatment and influence the likelihood for relapse [108]. In this regard, a recent study identified a selective enrichment of mutations in *CREBBP* and other epigenetic regulators (*SETD2*, *KDM6A*, *MLL2*, and *MSH6*) in relapsed and/or chemoresistant childhood B-ALL, which could suggest that epigenetic regulation plays a central role in clonal survival and ultimately chemotherapy resistance and relapse [97]. In addition to mutations in *CREBBP* and *SETD2*, relapse-specific mutations in the *NT5C2* gene have been described in childhood B-ALL [109,110]. NT5C2 is a 5’ nucleotidase responsible for the inactivation of nucleosidase-analog drugs, therefore conferring resistance to conventional therapeutic agents such as 6-mercaptopurine (6-MP) [109].

Although the true prognostic importance of these mutations at both diagnosis and relapse is unknown, therapies aimed at modulating epigenetic regulators in B-ALL is already in clinical development, as mentioned previously. Interstingly, CREBBP has also been shown to play a role in Wnt/β-catenin signaling, a pathway critical for the self-renewal of normal hematopoietic progenitor cells. In this regard, ICG-001, a novel small-molecule modulator of Wnt/β-catenin signaling that binds to CREBBP, leads to the differentiation of pre-B ALL cells and loss of self-renewal capacity, thereby sensitizing cells to chemotherapeutic treatment [111].

## Conclusions

Advances in genetic technologies have enriched our current understanding of childhood B-ALL. Although conventional karyotyping and FISH technologies play a significant role in detecting numerous recurrent abnormalities, microarray-based techniques and NGS have revealed a multitude of new molecular targets that may prove useful in the diagnosis, risk stratification, and most importantly individualized treatment of this disease.

## Consent

Written informed consent was obtained from the patient’s guardian/parent/next of kin for the publication of this report and any accompanying images.

## Competing interests

The authors declare that they have no competing interests.

## Authors’ contributions

All authors wrote, reviewed and approved the manuscript.
